# Epidemiological and Serological Investigation into the Role of Gestational Maternal Influenza Virus Infection and Autism Spectrum Disorders

**DOI:** 10.1128/mSphere.00159-17

**Published:** 2017-06-21

**Authors:** Milada Mahic, Xiaoyu Che, Ezra Susser, Bruce Levin, Ted Reichborn-Kjennerud, Per Magnus, Camilla Stoltenberg, Lokendrasingh Chauhan, Thomas Briese, Michaeline Bresnahan, Pål Surén, Mady Hornig, Siri Mjaaland, W. Ian Lipkin

**Affiliations:** a Center for Infection and Immunity, Mailman School of Public Health, Columbia University, New York, New York, USA; b Norwegian Institute of Public Health, Oslo, Norway; c Department of Epidemiology, Mailman School of Public Health, Columbia University, New York, New York, USA; d New York State Psychiatric Institute, New York, New York, USA; e Department of Biostatistics, Mailman School of Public Health, Columbia University, New York, New York, USA; f Department of Global Public Health and Primary Care, University of Bergen, Bergen, Norway; g KG Jebsen Center for Influenza Vaccine Research, Oslo, Norway; University of Michigan—Ann Arbor

**Keywords:** autism, birth cohort, infection, influenza virus, prenatal, serology

## Abstract

The literature concerning gestational maternal influenza virus infection and risk of autism spectrum disorders (ASD) is inconclusive. To address this uncertainty, we obtained information from questionnaires and samples from the Autism Birth Cohort, a prospective birth cohort comprising mothers, fathers, and offspring recruited in Norway in 1999 to 2008. Through questionnaires, referrals, and linkages to the Norwegian National Patient Registry, we identified 338 mothers of children with ASD and 348 frequency-matched controls for whom plasma samples that had been collected midpregnancy and after delivery were available for influenza virus serology via luciferase immunoprecipitation and hemagglutinin inhibition assays for influenza virus strains circulating during the study period. Assay data were combined to define serological status and integrated with self-reports of influenza-like illness to estimate ASD risk. Neither influenza A nor influenza B virus infection was associated with increased ASD risk. Integration of reports of symptoms of influenza-like illness with serology revealed an increase in risk for seropositive women with symptoms, but this increase did not achieve statistical significance (a level of *P* < 0.05) in the comparison with seronegative women without symptoms (adjusted odds ratio, 1.93; 95% confidence interval, 0.95 to 3.89; *P* = 0.068). Although chance may explain our findings, the magnitude of the potential association may be of biological importance, and dismissing our findings could result in failure to detect a bona fide association (type II error). If the association is true, we posit that the risk is due to activation of the maternal immune system following infection rather than direct fetal infection. Data on levels of cytokines or other mediators of inflammation would allow us to test the validity of this hypothesis.

**IMPORTANCE** The causes of most cases of autism spectrum disorders (ASD) are unknown. Some epidemiological studies suggest that maternal gestational influenza virus infection may increase the risk of ASD in offspring. Here, we describe an analysis of a large birth cohort with results based on questionnaires that prospectively addressed subjective reports of influenza-like illness and serological assays for objective determination of influenza virus infection. Although serologic evidence of gestational influenza virus infection alone was not associated with risk, positive serology and symptoms of influenza-like illness cannot yet be definitely ruled out as a risk factor.

## Abstract (precis)

The causes of most cases of autism spectrum disorders (ASD) are unknown. Some epidemiological studies suggest that maternal gestational influenza virus infection may increase the risk of ASD in offspring. Here, we describe an analysis of a large birth cohort with results based on questionnaires that prospectively addressed subjective reports of influenza-like illness and serological assays for objective determination of influenza virus infection. Although serologic evidence of gestational influenza virus infection alone was not associated with risk, positive serology and symptoms of influenza-like illness cannot yet be definitely ruled out as a risk factor.

## INTRODUCTION

Autism spectrum disorders (ASD), a heterogeneous group of neurodevelopmental disorders characterized by communication and social interaction impairments, restricted interests, and repetitive behaviors, have an estimated global prevalence of up to 2% (1) and an annual economic cost in the United States alone of $236 billion (2). The pathogenesis of ASD is poorly understood; there are no biomarkers for diagnosis, and treatment is largely supportive. Epidemiological studies and research in animal models suggest that both genetic and environmental factors have a role in disease (3–5). We recently reported that boys born to women with high titers of serum antibodies to herpes simplex type 2 at midpregnancy had an increased risk of subsequent diagnosis of ASD and speculated about a mechanism whereby infection triggers innate immune responses that result in neurodevelopmental damage (6).

Influenza viruses annually infect 5 to 15% of the global population (7). Most individuals have mild disease; however, high-risk groups, such as the young, elderly persons, and chronically ill people, may develop complications that include respiratory failure and death (8). Approximately 30,000 people die annually in the United States alone of influenza complications (9). Pregnant women infected with influenza viruses are also at increased risk for complications, including preterm labor, preterm delivery, and birth defects (10). Whether influenza can be linked to human neurodevelopmental disorders is less clear. The biological plausibility of this linkage has been established in animal models of gestational influenza (11). Influenza virus infection has been linked through serology to schizophrenia in epidemiological analyses (12); however, the only evidence linking gestational influenza virus infection to ASD risk has been based on self-reported data or medical records (13–15).

Here, we assess the risk of ASD in children born to mothers with serologically confirmed influenza virus infection during pregnancy.

## RESULTS

The study included plasma samples taken at two points during pregnancy, midpregnancy and after delivery, from 338 mothers of children with ASD and 348 mothers of control children without an ASD diagnosis. Characteristics of the study sample are shown in Table 1. Mothers of ASD children were more likely to be first-time mothers.

**TABLE 1:** Distribution of characteristics among study subjects

Characteristic	No. (%) in cohort		*P* value
	ASD	Control	
Total no. of mothers in study	338	348	
Maternal characteristics			
Maternal age (yr)			0.561
<25	53 (15.7)	45 (12.9)	
25–29	110 (32.5)	105 (30.2)	
30–34	121 (35.8)	138 (39.7)	
≥35	54 (16.0)	60 (17.2)	
Education (yr)			0.518
<12	40 (11.8)	32 (9.2)	
12	103 (30.5)	112 (32.2)	
13–16	113 (33.4)	116 (33.3)	
≥17	53 (15.7)	66 (19.0)	
No data	29 (8.6)	22 (6.3)	
Employment status			0.490
Employed/student	268 (79.3)	293 (84.2)	
Unemployed/benefits	36 (10.7)	33 (9.5)	
No data	34 (10.1)	22 (6.3)	
Smoked during pregnancy			0.495
No	271 (80.2)	296 (85.1)	
Yes	41 (11.8)	44 (13.0)	
No data	23 (6.8)	11 (3.2)	
Parity			0.040
0	176 (52.1)	162 (47.9)	
≥1	154 (44.3)	194 (55.7)	
Living status			0.988
Married/cohabiting	297 (87.9)	315 (90.5)	
Single/other	15 (4.4)	16 (4.6)	
No data	26 (7.7)	17 (4.9)	
Child’s characteristics			0.316
Sex			
Boys	281 (83.1)	279 (80.2)	
Girls	57 (16.9)	69 (19.8)	
Gestational age at sample collection^a^			
Midpregnancy (14–39 wks)	18.30 (1.65)	18.23 (1.48)	
Postpartum (27–43 wks)	39.31 (2.30)	39.60 (1.68)	

a Data for gestational age are mean values (with standard deviations in parentheses).

Results of luciferase immunoprecipitation system (LIPS) assays demonstrated that the majority of influenza virus infections were due to influenza A virus (35% of cases, 34% of controls) rather than influenza B virus (21% of cases, 27% of controls). Influenza A and influenza B viral infections were more frequent prior to conception or in early pregnancy than in late pregnancy. The frequency of infections did not differ significantly between cases and controls. Neither influenza A nor influenza B virus infection during pregnancy was associated with increased risk of ASD, regardless of the timing of the infection (Table 2). As verified with hemagglutination inhibition (HI) assays, the majority of women with influenza virus infection were exposed to H3N2 viruses, the virus type most prevalent during the 1999 to 2008 seasons (see Fig. S1 and Table S1 in the supplemental material).

10.1128/mSphere.00159-17.1 FIG S1  Number of women who were pregnant during the study period, stratified by influenza season. Download FIG S1, PDF file, 0.03 MB. Copyright © 2017 Mahic et al. 2017 Mahic et al. This content is distributed under the terms of the Creative Commons Attribution 4.0 International license.

10.1128/mSphere.00159-17.2 TABLE S1  Circulating influenza virus isolates during the study period (1999 to 2008) in Norway. (Ed. note: a previously posted version of this table was incorrect and was replaced on 20 July 2017 with the correct version.) Download TABLE S1, XLSX file, 0.01 MB. Copyright © 2017 Mahic et al. 2017 Mahic et al. This content is distributed under the terms of the Creative Commons Attribution 4.0 International license.

**TABLE 2:** Serologically confirmed maternal exposure to influenza virus in pregnancy and risk of ASD

Virus type and exposure group	No. (%) in cohort		OR (95% CI)	P value
	ASD	Non-ASD		
Both virus types				
Unexposed	170 (55.0)	177 (54.8)	1.0^a^	
Any time during pregnancy	139 (45.0)	146 (45.2)	0.99 (0.73–1.36)	0.956
Preconception/early pregnancy	111 (35.9)	109 (33.7)	1.06 (0.76–1.49)	0.734
Late pregnancy	34 (11.0)	43 (13.3)	0.82 (0.50–1.35)	0.443
Influenza A virus				
Unexposed	175 (65.1)	175 (66.5)	1.0^a^	
Any time during pregnancy	110 (34.6)	112 (33.5)	1.05 (0.76–1.46)	0.755
Preconception/early pregnancy	84 (26.4)	82 (24.5)	1.10 (0.77–1.57)	0.608
Late pregnancy	26 (8.2)	30 (9.0)	0.93 (0.53–1.62)	0.796
Influenza B virus				
Unexposed	252 (79.0)	242 (73.5)	1.0^a^	
Any time during pregnancy	67 (21.0)	87 (26.5)	0.74 (0.51–1.07)	0.104
Preconception/early pregnancy	58 (18.2)	70 (21.3)	0.80 (0.54–1.18)	0.251
Late pregnancy	9 (2.8)	17 (5.2)	0.51 (0.22–1.16)	0.109

a Reference group.

The final study group included 309 mothers of ASD offspring and 323 mothers with non-ASD offspring for whom we had sufficient plasma to complete HI assays for all relevant influenza virus strains. The proportion of mothers with serologically confirmed influenza virus infection during pregnancy was 45% for cases and controls, with both groups being more frequently seropositive around conception or in early pregnancy versus late pregnancy. No association was found between serologically confirmed influenza virus infection and ASD.

Symptoms of influenza-like illness were reported in 19% of case mothers and 14% of control mothers. A modest trend toward increased risk was observed among women with symptoms; however, the association was not statistically significant (adjusted odds ratio [aOR], 1.49; 95% confidence interval [CI], 0.95 to 2.35; *P* = 0.085).

Integration of reports of symptoms of influenza-like illness with serology records revealed an increase in risk of ASD in seropositive women with symptoms (aOR, 1.93; 95% CI, 0.95 to 3.89; *P* = 0.068) compared to seronegative women without symptoms (Table 3). The association was not statistically significant, especially not after adjustment for multiple testing. Measures of interaction between serologically confirmed maternal influenza and self-reported symptoms of influenza-like illness suggested that the symptoms of influenza-like illness could be effect modifiers of influenza virus infection, but the test results did not provide strong statistical evidence (relative excess risk [RERI] due to interaction on additive scale = 0.95 [95% CI, −0.49 to 2.40, *P* = 0.195]; ratio of odds ratios [ORs] on multiplicative scale = 2.01 [95% CI, 0.78 to 2.40, *P* = 0.146]).

**TABLE 3:** Risk of ASD in children prenatally exposed to influenza virus

Time of exposure during pregnancy	Exposure group	No. (%) in group^a^		OR (95% CI); *P* value	aOR (95% CI); *P* value
		ASD	Non-ASD		
Any time during pregnancy^b^	Sero_neg _ILI_neg_	140 (45.3)	149 (46.1)	1.0^c^	1.0^c^
	Sero_neg _ILI_pos_	30 (9.7)	29 (9.0)	1.12 (0.62–2.04); 0.700	1.11 (0.61–2.04); 0.733
	Sero_pos _ILI_neg_	112 (36.2)	131 (40.6)	0.90 (0.64–1.28); 0.571	0.86 (0.60–1.23); 0.409
	Sero_pos _ILI_pos_	27 (8.7)	15 (4.6)	1.94 (0.98–3.87); 0.059	1.93 (0.95–3.89); 0.068
Preconception/early pregnancy	Sero_neg _ILI_neg_	183 (59.2)	200 (61.9)	1.0^c^	1.0^c^
	Sero_neg _ILI_pos_	15 (4.9)	14 (4.3)	1.16 (0.53–2.55); 0.718	1.15 (0.51–2.59); 0.735
	Sero_pos _ILI_neg_	100 (32.4)	101 (31.3)	1.08 (0.77–1.52); 0.663	1.03 (0.72–1.47); 0.869
	Sero_pos _ILI_pos_	11 (3.6)	8 (2.5)	1.51 (0.59–3.86); 0.387	1.49 (0.58–3.87); 0.408
Late pregnancy	Sero_neg _ILI_neg_	247 (79.9)	259 (80.2)	1.0^c^	1.0^c^
	Sero_neg _ILI_pos_	28 (9.1)	21 (6.5)	1.43 (0.77–2.67); 0.260	1.44 (0.77–2.72); 0.256
	Sero_pos _ILI_neg_	28 (9.1)	40 (12.4)	0.73 (0.44–1.23); 0.238	0.72 (0.42–1.22); 0.223
	Sero_pos _ILI_pos_	6 (1.9)	3 (0.9)	2.06 (0.51–8.36); 0.312	2.18 (0.53–9.04); 0.282

a The total numbers of children in the ASD and non-ASD groups were 309 and 323, respectively.

b Timing of Sero and ILI during pregnancy was not concordant for all mothers in the study.

c Reference group.

Sensitivity analyses were conducted to test if the observed trend in increased risk among seropositive individuals with symptoms of influenza-like illness was dependent on the severity of symptoms. No association between influenza-like illness severity and ASD risk was observed; however, the group with seropositive individuals with severe influenza-like illness was small, resulting in wide confidence intervals (data not shown).

## DISCUSSION

This study was undertaken to investigate the potential association between serologically confirmed gestational influenza virus infection and ASD. Our data do not support an association between ASD and either gestational influenza or influenza-like illness in isolation, but they do suggest that the risk may be increased in instances where mothers have bona fide infection with an influenza-like illness.

We used two independent testing platforms, LIPS and HI, to analyze maternal plasma samples taken at two points during pregnancy (midpregnancy and after delivery) in order to assess serological status related to influenza virus infection, and we combined these objective findings with self-reported symptoms of influenza-like illness. The rates of seasonal influenza depend on many factors, such as duration of the season, previous virus exposure, pathogenicity of the circulating virus, and vaccination, and the reported rates range from 2% to 22% during the second and third trimesters (16). In our study, the overall influenza incidence was 45% (influenza A virus, 35% of cases and 34% of controls; influenza B virus, 21% of cases and 27% of controls), with a higher proportion of both case and control mothers infected prior to conception and in early pregnancy rather than in late pregnancy. Differences in infection rates between the preconception/early pregnancy and late pregnancy groups may simply reflect a longer observational interval for the first group. In addition, since exposure status for preconception/early pregnancy was determined using a single antibody titer, some mothers could have been misclassified if they were recently infected with virus strains showing cross-immunoreactivity with a tested strain(s).

We found a higher incidence of influenza virus infection than previously reported for pregnant women. Some mothers in our study were pregnant during two influenza seasons, and all individuals, with or without symptoms, were tested for both influenza A and influenza B virus infection. This, in addition to possible nondifferential misclassification of early exposure, may have led to our finding higher infection rates.

All influenza seasons during our study period were dominated by influenza A viruses, as reflected by a higher prevalence of influenza A than influenza B virus infections for all mothers. We frequency matched the cases and controls by birth year to minimize the impact of differences in pathogenicity of circulating viruses; however, numbers of women exposed during different seasons were too low to address whether risk was strain specific. Information on symptoms of influenza was collected prior to ASD diagnosis, thereby minimizing the potential for retrospective bias.

Previous studies into the association between seasonal influenza virus infection and ASD were based on self-reported symptoms or information from medical records (13–15). Influenza virus infection was not serologically confirmed in these studies; thus, exposure estimates were likely inaccurate. The main strengths of our study are that we had serological measures of influenza virus infection from maternal samples collected during pregnancy and at delivery, that influenza-like illness was reported by mothers during and after pregnancy prior to diagnosis of ASD in their children, and that the ASD diagnosis was obtained from the Norwegian patient registry. The main weakness of our study is the limited sample size, which did not allow for greater certainty in our estimates.

Influenza virus triggers an array of acute-phase responses, including fever and systemic increased cytokine expression (17, 18). Both animal laboratory and human epidemiological studies suggest that hyperthermia is associated with an increased risk for adverse neurodevelopmental outcomes, especially neural tube defects (19). We did not obtain cytokine measurements; however, all mothers who reported influenza-like illness also reported fever, which may serve as a proxy for proinflammatory cytokines (20).

Epidemiological data suggest that admission to the hospital for maternal viral infection in the first trimester or maternal bacterial infection in the second trimester is associated with increased risk of ASD (21, 22). A recent study reported a potential link between influenza vaccination in the first trimester and ASD (15). In addition, analysis of cytokine levels in maternal midpregnancy samples suggested an association between increased proinflammatory cytokines and ASD risk, but these findings were restricted to ASD individuals with the comorbidity of intellectual disability (23).

We did not find variations in risk based on the timing of infection in our study. This could be explained by our small sample size, lack of information on influenza-like illness before conception, and imprecision in the timing of self-reported influenza-like illness and serology.

In conclusion, we report here a potential association between symptomatic influenza virus infection and ASD that is not strongly supported statistically, especially after adjustment for multiple testing. We cannot rule out chance as a possible explanation of the findings; however, the magnitude of the potential association in terms of ORs may be of biological importance. Dismissing these findings could result in failure to detect a bona fide association (type II error). If the association is true, we posit that the increased risk may be due to activation of the maternal immune system following infection rather than direct fetal infection. Data on the levels of cytokines or other mediators of inflammation would allow us to test the validity of this hypothesis.

## MATERIALS AND METHODS

### Study subjects.

The Autism Birth Cohort (ABC) study is a case-control study nested within the Norwegian Mother and Child Cohort study (MoBa), which includes 114,479 children, 95,244 mothers, and 75,500 fathers who were recruited by the Norwegian Institute of Public Health (NIPH) from 1999 to 2008. Maternal blood samples were collected at pregnancy week 18 and after delivery, processed to extract plasma within 30 min, and stored at −80°C (24).

Children with ASD were identified through questionnaire screening of mothers at offspring ages of 3, 5, and 7 years, professional and parental referrals of participants suspected of having ASD, and annual linkages to the Norwegian Patient Register (NPR). A subset of children was diagnosed at the ABC clinic in Oslo. Our study group included 338 mothers of ASD cases and 348 control women from whom plasma samples obtained at midpregnancy and after delivery were available for serological analysis. Controls were frequency matched based on sex, birth year, and birth month. Multiple-gestation pregnancies were excluded.

### Serological analyses. (i) LIPS assay.

LIPS technology (25), based on luciferase-tagged antigens produced in mammalian cells, was used to screen the plasma samples for evidence of influenza virus infection by quantitating antibodies against influenza nonstructural protein 1 (NS 1). NS1 was selected as the target in these assays because it is only present if viral replication has taken place (26) and it is not induced by vaccination. pREN2, a mammalian *Renilla* luciferase (Ruc) expression vector, was used to generate NS1 fusion protein constructs. Six plasmid templates for NS1 from influenza A virus (four H3N2 and two H1N1 subtypes) and four from influenza B virus (two from Victoria and two from Yamagata lineages) were amplified by PCR and subcloned into pREN2; the resulting constructs generated C-terminal fusions to Ruc (Table S1).

Extracts containing the Ruc-NS1 proteins for influenza A and influenza B virus fusions were prepared from transfected Cos1 cells (27). Specificity for influenza A virus and capacity to discriminate between NS1 of H3N2 and H1N1 viruses were tested for six influenza A virus fusion constructs. As positive controls, we used sera specific for seven different H1N1 strains, six H3N2 strains, one Victoria strain, and two Yamagata influenza virus strains. The constructs were demonstrated to be specific in detecting influenza A virus but did not discriminate between H3N2 and H1N1. Similar results were observed with influenza B virus constructs.

To define seropositivity, a simple statistically based cutoff was derived for each antigen from the mean value of the signal from uninfected samples plus 5 standard deviations. All samples with values higher than 50,000 light units (LU) were also classified seropositive.

The presence of anti-NS1 antibodies above cutoff levels was interpreted as an indication of either past or current infection. To estimate the timing of infection in relation to the pregnancy and to define specificity, all LIPS-positive samples were further tested in HI assays.

### (ii) HI assay.

We measured anti-hemagglutinin antibodies against seasonal influenza A and influenza B viruses circulating during our study period (Table S1). Each plasma sample was treated with receptor-destroying enzyme (RDE) prior to use. The HI assay was performed with a 0.5% turkey red blood cell suspension as described elsewhere (28). Viral isolates received from the Centers for Disease Control and Prevention and the World Health Organization were propagated in MDCK cells (29). Influenza B viruses were ether treated prior to use to increase assay sensitivity (30). All plasma samples were tested in pairs (midpregnancy sample and birth sample on the same plate), together with positive- and negative-control plasma samples.

Twenty different strains of seasonal influenza A and influenza B viruses circulated in Norway during the inclusion period for MoBa participants (1999 to 2008). Each mother was tested for exposure to the viral strains circulating during her pregnancy. If more than one influenza virus strain was prevalent in the given period, or a woman was pregnant during two influenza seasons, a separate HI assay was conducted for each strain. The HI titer was read as the reciprocal of the highest serum dilution causing complete inhibition of agglutination. Partial agglutination was not scored as inhibition of agglutination. If there was no HI at the highest serum concentration (1:10 dilution), the titer was designated a 5. Antibody titers of ≥20 for influenza A virus and ≥40 for influenza B virus were considered indicative of infection. All samples with signs of inhibition of hemagglutination were repeated.

### Classification of individuals based on serological status.

Timing of serological infection in relationship to pregnancy was determined for all women who had tested positive on LIPS assays for the presence of anti-NS1 antibodies, based on the results of the subsequent HI assays (Table S2). Seroconversion between midpregnancy and delivery was defined as a 4-fold increase in HI titer or a change from being seronegative (<1:20) to seropositive (≥1: 20) between two samples for influenza A virus, and from <1:40 to a ≥1: 40 for ether-treated influenza B virus. Women who seroconverted were classified as influenza virus exposed in late pregnancy. Women who were HI positive at baseline (midpregnancy sample) but did not seroconvert between midpregnancy and birth were classified as influenza virus exposed during the preconception/early pregnancy period if there was an overlap between seasonal influenza epidemics and the early part of pregnancy. Early pregnancy was defined as time from conception to midpregnancy (sampling time for the first plasma sample). Timing of influenza epidemics for every season was defined by the Influenza Surveillance Centre at the Norwegian Institute of Public Health as periods when the frequency of weekly reported influenza-like illness from primary health care providers was above the threshold of 1.5%.

10.1128/mSphere.00159-17.3 TABLE S2  Maternal exposure to influenza A and influenza B viruses at midpregnancy and birth, based on antibody levels measured via LIPS and HI assays. HI assays were performed for samples that were LIPS positive at either midpregnancy or birth. Samples were classified as LIPS positive if they had levels of anti-NS1 antibodies above the cutoff value for seropositivity. Download TABLE S2, PDF file, 0.1 MB. Copyright © 2017 Mahic et al. 2017 Mahic et al. This content is distributed under the terms of the Creative Commons Attribution 4.0 International license.

### Definition of influenza-like illness based on questionnaire data.

Data on selected maternal symptoms during pregnancy were collected from three questionnaires answered by participants in the MoBa study (31). Pregnant women who agreed to participate completed the first questionnaire during gestational weeks 13 to 17 (Q1). They answered the second questionnaire during gestational weeks 30 to 33 (Q3) and the third questionnaire when the child was 6 months old (Q4). In all questionnaires, the pregnant woman was asked to indicate by checking a box whether she had experienced any of several symptoms or used medications for those symptoms during the current pregnancy. She was also asked to indicate the gestational week interval in which she had had the symptoms (Q1, weeks 0 to 4, 5 to 8, 9 to 12, and 13; Q3, weeks 13 to 16, 17 to 20, 21 to 24, 25 to 28, and 29+; Q4, last part of pregnancy). In this study, symptoms reported from weeks 1 through 20 were classified as early pregnancy and those from week 21 to delivery were classified as late pregnancy. For the case definition of influenza-like illness, the following criteria were applied: for Q1, fever and common cold, influenza, or throat infection; Q3, fever and common cold or influenza or throat infection or other cough; Q4, fever and common cold/influenza or sore throat/sinusitis/ear infection.

Sensitivity analyses were conducted for severity of influenza-like illness, with severe cases defined as reporting fever and common cold or influenza and respiratory symptoms (throat infection or cough).

### Covariates.

Variables potentially influencing both the risk of influenza and the risk of ASD were identified as possible confounders. Measures of association (odds ratios) were adjusted for maternal age, parity, and education, in addition to the matching variables (birth year and sex of the child).

### Statistical analysis.

Data were analyzed using IBM SPSS Statistics for Windows, version 23.0. (IBM Corp., Armonk, NY), MatLab and Statistics Toolbox release 2013a (MathWorks, Inc., Natick, MA), and RStudio, running R version 3.3.1 (RStudio, Inc., Boston, MA). Characteristics of controls and cases were compared using chi-squared tests. Binary logistic regression was applied to estimate crude and adjusted ORs of ASD in the offspring, with associated 95% CIs. To test if the risk depended on the symptoms, we tested for additive and multiplicative interactions between serology and self-reported influenza-like illness. Multiplicative interactions between serology and symptoms of influenza-like illness were assessed by including the interaction term in the regression analyses. For quantification of additive interactions, the relative excess risk due to interaction (RERI) was calculated as recommended in the literature, and the associated 95% CI was calculated using the delta method (32).

The Markov chain Monte Carlo (MCMC) multiple-imputation method (33) with 50 repeated imputations was applied to complete missing influenza-like illness values for 111 serologically tested mothers as well as for missing values in covariates. Rubin’s formula (34) was used for combining multiple estimates. Medians were used when proportions were calculated. A statistical significance level of 0.05 was used for all analyses; reported *P* values are based on two-tailed tests.
