# Supplementary figures and images for: Epidemiological and Serological Investigation into the Role of Gestational Maternal Influenza Virus Infection and Autism Spectrum Disorders
Source: mSphere. 2017 Jun 21;2(3):e00159-17. doi: 10.1128/mSphere.00159-17 (PMC5480032; doi:10.1128/mSphere.00159-17)

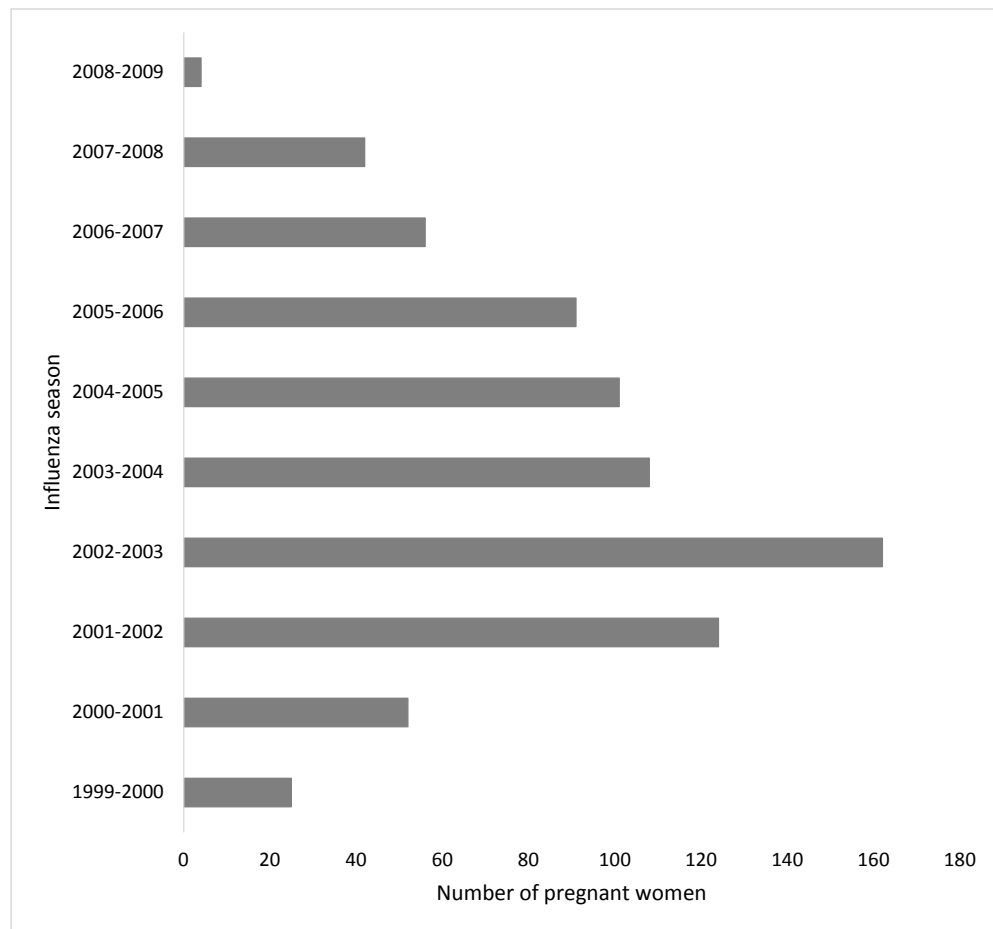

Supplement: FIG S1 [file sph003172300sf1.pdf]
