# Supplementary material for: Epidemiological and Serological Investigation into the Role of Gestational Maternal Influenza Virus Infection and Autism Spectrum Disorders
Source: mSphere. 2017 Jun 21;2(3):e00159-17. doi: 10.1128/mSphere.00159-17 (PMC5480032; doi:10.1128/mSphere.00159-17)
Supplement: TABLE S2 [file sph003172300st3.pdf]

Supplemental Table S2

			Mid-pregnancy	Birth		Mid-pregnancy	Birth
Influenza A	ASD	LIPS negative	61 (19.2 %)	65 (20.4 %)			
		LIPS positive*	257 (80.8 %)	253 (79.6 %)	HI negative	106 (40.0 %)	86 (32.5 %)
					HI positive	159 (60.0 %)	179 (67.5 %)
		Total	*318*	*318*		*265*	*265*
	non-ASD	LIPS negative	69 (20.6 %)	69 (20.6 %)			
		LIPS positive*	266 (79.4 %)	266 (79.4 %)	HI negative	111 (39.9 %)	87 (31.3 %)
					HI positive	167 (60.1 %)	191 (68.7 %)
		Total	*335*	*335*		*278*	*278*
Influenza B	ASD	LIPS negative	175 (54.9 %)	185 (58.0 %)			
		LIPS positive*	144 (45.1 %)	134 (42.0 %)	HI negative	31 (20.5 %)	22 (14.6 %)
					HI positive	120 (79.5 %)	129 (85.4 %)
		Total	*319*	*319*		*151*	*151*
	non-ASD	LIPS negative	161 (48.9 %)	169 (51.4 %)			
		LIPS positive*	168 (51.1 %)	160 (48.6 %)	HI negative	43 (23.9 %)	23 (12.8 %)
					HI positive	137 (76.1 %)	157 (87.2 %)
		Total	*329*	*329*		*180*	*180*

* Samples that were LIPS positive at either mid-pregnancy or birth were further tested with HI assays

ASD: Autism Spectrum Disorder
